# Objective evaluation of physical activity pattern using smart devices

**DOI:** 10.1038/s41598-019-38638-z

**Published:** 2019-02-14

**Authors:** Monika Šimaitytė, Andrius Petrėnas, Julija Kravčenko, Eleni Kaldoudi, Vaidotas Marozas

**Affiliations:** 1 Biomedical Engineering Institute, Kaunas University of Technology, Kaunas, Lithuania; 2 Faculty of Electrical and Electronics Engineering, Kaunas University of Technology, Kaunas, Lithuania; 3 Medical Imaging and Telemedicine, School of Medicine, Democritus University of Thrace, Dragana, Alexandroupoli Greece

## Abstract

Physical activity session frequency and distribution over time may play a significant role on survival after major cardiovascular events. However, the existing amount-based metrics do not account for these properties, thus the physical activity pattern is not fully evaluated. The aim of this work is to introduce a metric which accounts for the difference between the actual and uniform distribution of physical activity, thus its value depends on physical activity aggregation over time. The practical application is demonstrated on a step data from 40 participants, half of them diagnosed with chronic cardiovascular disease (CVD). The metric is capable of discriminating among different daily patterns, including going to and from work, walking in a park and being active the entire day. Moreover, the results demonstrate the tendency of CVD patients being associated with higher aggregation values, suggesting that CVD patients spend more time in a sedentary behaviour compared to healthy participants. By combining the aggregation with the intensity metric, such common weekly patterns as inactivity, regular activity and “weekend warrior” can be captured. The metric is expected to have clinical relevance since it may provide additional information on the relationship between physical activity pattern and health outcomes.

## Introduction

The impact of physical activity pattern on health outcomes is an ongoing debate and has recently received considerable research attention^1–6^. It has been hypothesized, that long-term monitoring and pattern correction with respect to physical activity session frequency and distribution over time may facilitate rehabilitation and lead to a reduced number of deaths, caused by cardiovascular diseases^7,8^. This reasoning can be supported by the reported association between regularly and irregularly performed physical activity and survival after major cardiovascular events, i.e., myocardial infarction^1^, and stroke^9^. The majority of studies for assessing the association of physical activity and health outcomes involve metrics that rely only on the amount of physical activity, of which the step counts, energy expenditure, and a time spent in physical activity of a particular intensity are the most commonly used^8,10,11^. Since the conventional amount-based metrics do not account for physical activity session distribution over time, the pattern is not fully evaluated. Therefore, it is desirable to develop novel approaches which may offer additional information on physical activity beyond what is provided by existing metrics^6,9,12^.

A few attempts have been made towards describing the physical activity pattern quantitatively^13–16^. Physical activity inequality quantified by the Gini coefficient was found to be a better predictor of obesity prevalence than the average number of steps^15^. Yet the intensity gradient, developed to describe the physical activity intensity distribution, showed an independent relationship with an average acceleration and was found to be associated with physical function in adults with type 2 diabetes^16^. These metrics provide different information than the amount-based metrics, however, they disregard the temporal distribution of physical activity sessions.

Devices with an embedded accelerometer (e.g., smart wristbands, smartphones) are becoming increasingly popular for use in physical activity research^15,17–19^. Most of them provide various activity-related information via cloud services or apps, which may increase motivation to enhance the physical activity level^20^. Nevertheless, a comprehensive evaluation of physical activity pattern that covers physical activity session frequency and distribution is still achieved using self-report data, such as logs, questionnaires, surveys and interviews^2,3,6,21^. These subjective methods are inconvenient for both the patient and the physician, and are less suitable for long-term monitoring (months, years) due to errors and recall bias^21^. Therefore objective monitoring using a more advanced technology is highly desirable.

In this paper, we propose a novel metric for quantifying physical activity patterns acquired by smart devices. In contrast to the existing approaches, the proposed metric accounts for a temporal distribution of physical activity sessions, and, when combined with conventional metrics (e.g., intensity), it can capture commonly observed patterns as inactivity, regular activity and “weekend warrior”^22^. The proposed metric was evaluated against existing metrics using synthesized step patterns, and was also applied to study activity behaviour of healthy participants as compared to cardiovascular disease (CVD) patients.

## Methods

In this study, physical activity is defined as a number of steps, accumulated over a time interval, e.g., 1 min, whereas physical activity pattern refers to the temporal distribution of steps over the monitoring period, e.g., during a day or week. It should be noted, that the metric proposed in this study is not restricted to the step data, but can also be applied for other types of physical activity such as energy expenditure, burned calories, etc.

### Study population

Accumulated minute-by-minute step data were obtained using a Fitbit Charge 2 (Fitbit, San Francisco, CA, the US) smart wristband from two groups of participants. Step data of the first group were used to synthesize commonly encountered physical activity patterns, in order to create a reference activity data set to evaluate the performance of the proposed metric. Meanwhile, step data of the second group were acquired during free-living activities, and were used to demonstrate a practical application of the proposed metric to study differences in activity patterns.

The first group consisted of 11 healthy participants (7 women), 20.0 ± 1.6 years old, with a body mass index of 21.7 ± 2.4 kg/m^2^. This group was instructed to perform standardized physical activities, namely, slow walking, fast walking and running. Step data collected were used to synthesize the most commonly encountered patterns (more details are provided in the subsection “Synthesized physical activity patterns”).

The second group consisted of 40 participants (24 women), 50.4 ± 14.6 years old, with a body mass index of 26.3 ± 4.8 kg/m^2^. The participants were recruited on a volunteer basis and randomly selected amongst patients who visited the primary health care clinic Signata (Kaunas, Lithuania) during the fourth quarter of 2017. Volunteers were assigned to the groups of 20 healthy participants and 20 CVD patients following the standard clinical protocol examination. CVD patients were diagnosed with chronic cardiovascular diseases, namely, ischemic heart disease, chronic heart failure and persistent atrial fibrillation. The participants were instructed to wear the smart wristband for at least seven days, except when showering, bathing or swimming, and were asked to maintain their usual physical activity regimens. Two out of 40 enrolled participants discontinued the monitoring after the six days, resulting in 278 days of total wear time (198 weekdays and 80 weekends). A signed, written consent to participate in a study was obtained from all the participants, and the principles of the Declaration of Helsinki were followed. Identifiable information was removed to ensure participant anonymity.

The data were originally collected by the information technology company Kvantas (Kaunas, Lithuania) and the primary health care clinic Signata during the project “An expert system for health risk profile assessment”, partially supported by the Lithuanian Business Support Agency (LBSA) under the Intellect LT measure (Agreement No. J05-LVPA-K-01-0254). Biomedical Engineering Institute of Kaunas University of Technology was subcontracted by the company Kvantas to process the data and develop the method for objective evaluation of physical activity pattern.

### Parametrization of physical activity pattern

**Figure 1:**
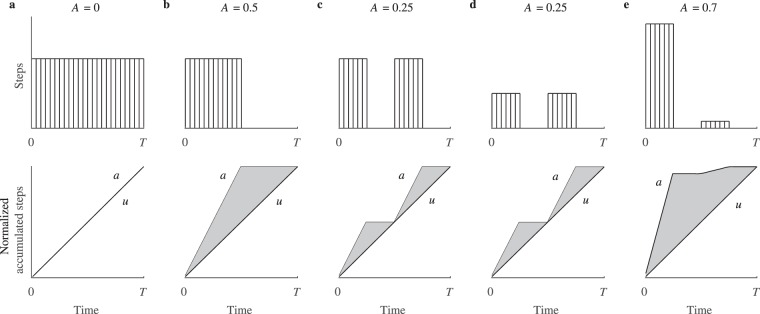
Graphical illustration of the computed physical activity aggregation *A* for various activity patterns. Physical activity patterns (above) with corresponding normalized accumulated steps (below) of actual (*a*) and reference uniform (*u*) distributions: (**a**) an evenly spread physical activity over the entire monitoring period, (**b**) a continuous single session with a duration of half of the total monitoring period, (**c**) two equal sessions of high intensity, (**d**) two equal sessions of low intensity, (**e**) two sessions of unequal intensity.

We propose a physical activity metric, namely, physical activity aggregation (*A*), that takes into account the step distribution over the monitoring period, thus its value depends on whether steps are evenly distributed over time or there are any intense activity sessions. Physical activity aggregation is defined as:

1 $$A=\frac{2}{SN}\sum _{i=1}^{N}|{a}_{i}-{u}_{i}|,$$

where *S* is the total number of steps during the monitoring period, *N* is the total number of time intervals, in which steps are accumulated, and *a*_*i*_ and *u*_*i*_ are the actual and uniform cumulative step distributions, respectively. The hypothetical uniform distribution represents evenly spread steps throughout the monitoring period, and serves as a reference for measuring the uniformity of the actual distribution (see the shaded area in Fig. 1).

The cumulative actual distribution *a*_*i*_ is calculated by

2 $${a}_{i,j+1}=\sum _{k=i}^{i+j}{s}_{k},i=\mathrm{1,}\ldots ,N-j,j=\mathrm{0,}\ldots ,N-\mathrm{1,}$$

3 $${a}_{i}={({a}_{i,j})}_{max},i,j=\mathrm{1,}\ldots ,N,$$

where *s* is the number of steps in time intervals (e.g., minute-by-minute step data).

The cumulative uniform distribution *u*_*i*_ is calculated by

4 $${u}_{i}=\frac{i}{N}\sum _{k=1}^{N}{s}_{k},i=\mathrm{1,}\ldots ,N\mathrm{.}$$

Physical activity aggregation *A* can take values between 0 and 1. Values close to 0 indicate low aggregation; this corresponds to physical activity patterns with steps evenly spread over the monitoring period (Fig. 1a). In contrast, values close to 1 indicate maximal temporal aggregation, inherent in patterns with a single continuous physical activity session, or with several sessions aggregated in a short time interval compared to the total monitoring period. It should be noted, that the aggregation metric depends on the duration of a physical activity session, i.e., *A* tends to be larger for shorter sessions. However, *A* is not affected by intensity, thus the same pattern, but with different intensity, would result in the same aggregation value (Fig. 1c and d).

Since no major activity is expected during the night, this period was excluded from the computation. The onset of the night was set when the number of steps per hour decreased to less than 20. Similarly, the end of the night was set when the number of steps per hour exceeded 20.

### Performance evaluation

The proposed metric was evaluated for its efficacy to describe and identify physical activity patterns via two sets of experiments: the first set involved synthesized data based on recordings of controlled physical activities performed by the first group of healthy participants; the second set of experiments involved demonstration of the metric to study free-living activities of healthy participants and CVD patient groups.

The proposed aggregation metric was compared with the physical activity intensity, which is commonly used as a threshold for moderate or vigorous physical activity^23,24^. Physical activity intensity *I* is defined as the average number of steps per time interval. The upper value of *I* is unlimited and depends on the time interval in which is accumulated. When steps are accumulated in the intervals of one minute, 100 steps is commonly considered as a moderate intensity physical activity^23^.

The Gini coefficient has been previously applied to evaluate inequality of physical activity within countries across the globe^15^. The Gini coefficient *G* is the ratio of the area that lies between the line of equality and the Lorenz curve to the total area under the line of equality^15,25^. The Gini coefficient takes values from 0 to 1, where 0 stands for the hypothetical uniform distribution (perfect equality) and values close to 1 are obtained for largely unequal physical activity distributions.

The statistical significance of the differences was determined using the Mann-Whitney U test, whereas the relationship between the aggregation metric and the metrics under comparison was assessed using the Spearman correlation coefficient.

### Synthesized physical activity patterns

**Figure 2:**
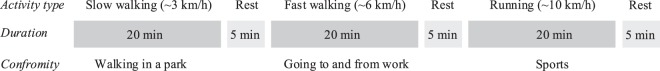
Acquisition of the standardized physical activities. Step data of these activities are further used to synthesize commonly encountered daily patterns.

The synthesized physical activity patterns were created to subjectively investigate the ability of the proposed aggregation metric to differentiate commonly encountered daily physical activity patterns. Standardized step data were obtained by asking the first group of participants to perform predetermined physical activities, each followed by a rest period (Fig. 2).

Using the acquired step data, four common daily patterns were synthesized according to the observations of physical activity patterns across free-living population worldwide, reported in^15,26^. Specifically, the most intensive physical activity is reported around 8 am and 6 pm on weekdays, whereas, activity is highly aggregated during mid-day on the weekends^15^. Accordingly, the patterns “going to and from work” and “walking in a park” have been synthesized. Two more patterns, namely “low intensity activity” and “going to/from work and sports”, were created to account for insufficiently active and very active individuals, respectively. Patterns were synthesized by placing steps of the standardized activity at the specific time, intrinsic for each pattern:

- The 1^st^ pattern represents insufficient physical activity over the entire day. Such pattern is intrinsic for individuals who spend a large amount of time in a sedentary behaviour and engage in low intensity physical activity. The pattern was obtained using daily steps from insufficiently active participant, extracted from long-term step recording. No standardized activity is added to this pattern.
- The 2^nd^ pattern simulated the activity of going to and from work. Such pattern is especially common among those working in an office. The pattern was synthesized using step data of fast walking placed at morning (8 am) and evening (6 pm) hours.
- The 3^rd^ pattern is identical to the 2^nd^, however, with additional activity of sports in the evening, after the workday. The sports activity was synthesized using step data recorded during running.
- The 4^th^ pattern represents walking in a park, which is a common activity during the weekend. It synthesized using step data acquired during slow walking placed at mid-day (from 1 pm to 2 pm).

**Figure 3:**
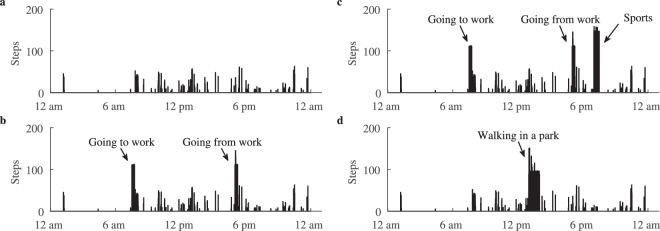
Synthesized physical activity patterns: (**a**) 1^st^ pattern represents low intensity physical activity over the entire day, (**b**) 2^nd^ pattern stands for the activity of going to and from work, (**c**) 3^rd^ pattern is identical to the 2^nd^, however, with additional activity of sports after the workday, and (**d**) 4^th^ pattern represents walking in a park, which is common activity on a weekend. Note that background low intensity activity is added to the 2^nd^, 3^rd^, and 4^th^ patterns.

To increase realism of the synthesized patterns, background low intensity physical activity, such as that in the 1^st^ pattern, was added to the 2^nd^, 3^rd^, and 4^th^ patterns. The synthesized dataset contains of a total of 11 × 4 = 44 daily patterns. Examples of synthesized patterns are shown in Fig. 3.

## Results

**Figure 4:**
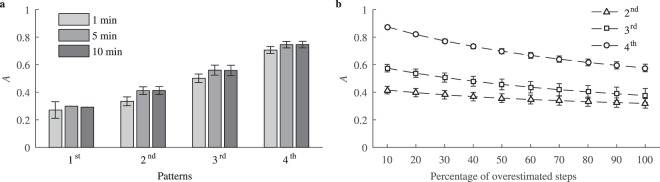
(**a**) Physical activity aggregation for synthesized physical activity patterns when steps are accumulated in a window of increasing duration: 1 min, 5 min, 10 min. (**b**) Influence of overestimated steps on *A*. Overestimated steps were synthesized by increasing the level of low intensity background activity. Results are expressed as mean ± two-sided 95% confidence interval.

The capability of the physical activity aggregation *A* to characterize distinct synthesized patterns is shown in Fig. 4. It is obvious that the patterns with highly aggregated physical activity, such as the 3^rd^ pattern, which refers to sports activity, and the 4^th^ pattern, which reflects walking in a park, take much larger values compared to the patterns with evenly spread physical activity. Smart devices may provide step data accumulated in a longer window than 1 min. The results show that *A* slightly increases for windows longer than 1 min; this observation applies to all the patterns under the investigation (Fig. 4a). Since the smart wristband is designed to be used on the arm, and not near the body mass center, monitoring may lead to extra steps counted due to arm movement. Figure 4(b) shows *A* as a function of a percentage of overestimated steps, synthesized by increasing the level of background low intensity physical activity. Aggregated physical activity is dominated by background activity, thus *A* decreases as the percentage of overestimated steps increases.

**Figure 5:**
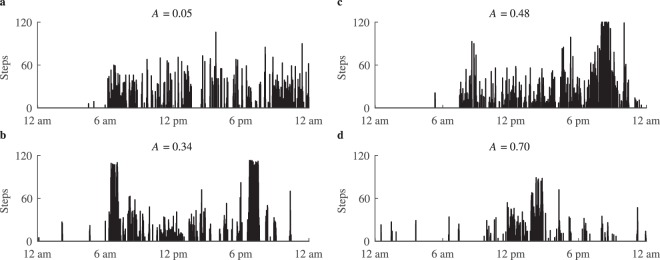
Physical activity patterns observed among CVD patients in a free-living condition: (**a**) uniformly distributed activity, (**b**) aggregated during the time periods corresponding to going to and from work, (**c**) aggregated during walking in the park at the evening, (**d**) most of the physical activity aggregated during the mid-day. Note that sleeping hours are excluded from computing the aggregation *A*.

The aggregation values computed for the free-living step data confirm that the proposed metric is well-suited for discriminating daily physical activity patterns (see Fig. 5). A low *A* value corresponds to patterns representing evenly spread physical activity, such as shown in Fig. 5(a). The pattern shown in Fig. 5(b) represents a case where steps are mostly aggregated during going to and from work. Similarly, the pattern in Fig. 5(c) represents an ordinary workday with additional 2 h of sports. Since half of the total physical activity is aggregated in the evening, the *A* value approaches 0.5. The example in Fig. 5(d) demonstrates the pattern of a sedentary behaviour with a short period of low intensity physical activity, therefore, almost all steps are aggregated in a single session, which produces a high physical activity aggregation value.

**Figure 6:**
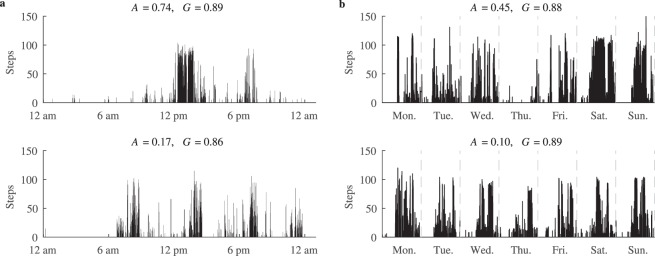
Examples of (**a**) daily and (**b**) weekly physical activity patterns of high (above) and low (below) aggregation. Note that the Gini coefficient is similar in all these cases.

Figure 6 shows comparatively the physical activity aggregation and the Gini coefficient for various activity patterns. The aggregation metric takes notably different values for distinct patterns, while the Gini coefficient is similar in all these particular cases. Moreover, the Gini coefficient is strongly negatively correlated with a simple metric, which defines the proportion of the total duration of physical activity to the total monitoring period (*r* = −0.98, *p* < 0.001). On the contrary, the aggregation metric is only moderately correlated with the Gini coefficient (*r* = 0.40, *p* < 0.001) and is weakly correlated with the intensity (*r* = 0.24, *p* < 0.001), demonstrating the potential of the proposed metric to provide independent information about the physical activity pattern.

**Figure 7:**
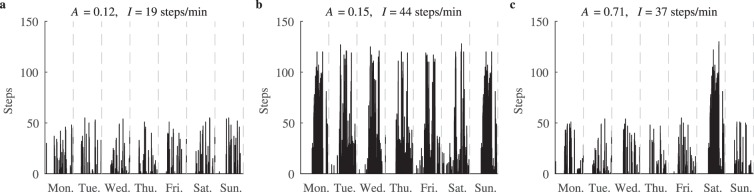
The most common weekly physical activity patterns: (**a**) inactivity, (**b**) regular activity and (**c**) the “weekend warrior” pattern, captured by the combination of the aggregation and intensity metrics.

Figure 7 shows the physical activity aggregation and activity intensity metrics for the three commonly encountered weekly activity patterns defined as: inactivity (no activity of moderate or vigorous intensity over the week), regular activity (more than three sessions of moderate or vigorous intensity), and “weekend warrior” (one or two sessions of moderate or vigorous intensity). The pattern of inactivity is characterized by low physical activity aggregation and low intensity values (Fig. 7a). Since the physical activity aggregation metric is not affected by the intensity, low aggregation and high intensity values suggest regular activity (Fig. 7b). Meanwhile, the “weekend warrior” pattern leads to a high physical activity aggregation value and a moderate intensity value (Fig. 7c).

**Figure 8:**
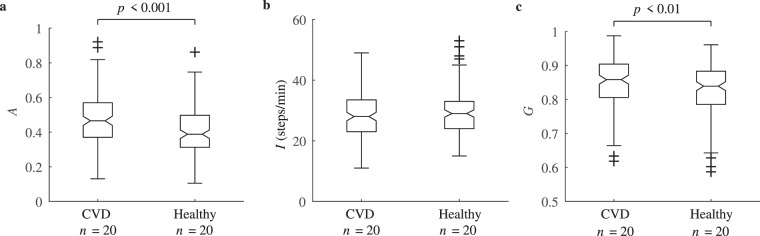
(**a**) Physical activity aggregation, (**b**) intensity and (**c**) Gini coefficient between the groups of CVD patients and healthy participants.

Figure 8 shows the physical activity aggregation, the activity intensity and the Gini coefficient for the groups of healthy participants and CVD patients. The median physical activity aggregation was 0.47 for CVD patients and 0.39 for healthy participants (*p* < 0.001). On the contrary, the intensity metric and the Gini coefficient for these groups do not produce statistically significant differences.

**Figure 9:**
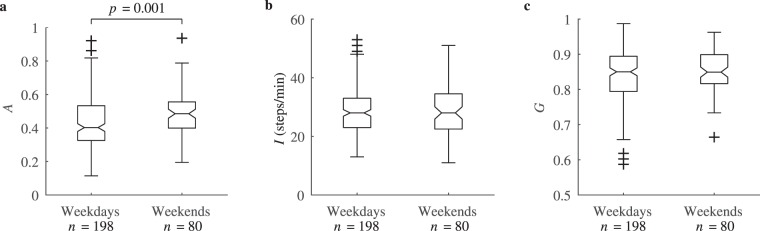
(**a**) Physical activity aggregation, (**b**) intensity and (**c**) Gini coefficient during weekdays and weekends.

Figure 9 shows that the median aggregation for weekend step data is about 20% larger than that for weekdays, being 0.49 and 0.40, respectively (*p* = 0.001). This can be explained by a single session of intensive aggregated physical activity, i.e., sports, walking or shopping, usually preferred on weekends. As previously, the activity intensity and the Gini coefficient presented no significant difference for the two population groups.

**Figure 10:**
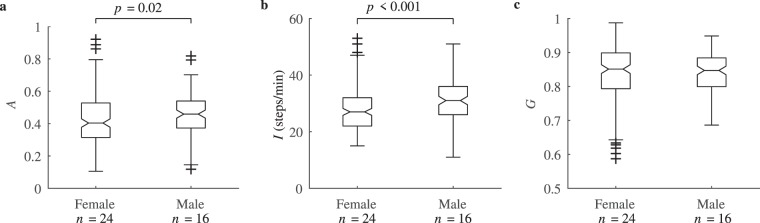
(**a**) Physical activity aggregation, (**b**) intensity and (**c**) Gini coefficient between the groups of female and male.

Figure 10 displays the physical activity aggregation, the activity intensity and the Gini coefficient for female and male participants. The results demonstrate the tendency of aggregation being lower for female than male (*p* = 0.02), suggesting females spend less time in a sedentary behaviour, although, males are associated with a higher intensity activity (*p* < 0.001).

## Discussion

This work introduces a metric for an objective evaluation of physical activity pattern which accounts for physical activity session frequency and distribution over time. While a positive effect on health has been observed for moderate physical activity of at least of 150 min weekly^12^, there is no consensus on the optimal distribution of physical activity sessions over time. That is, it is unclear whether 30 minutes of activity in 5 days or 50 minutes in 3 days are more beneficial for health. It is widely agreed that a regularly performed physical activity reduces the risk of cardiovascular disease and leads to an extended life expectancy^11,27–29^. A study on the influence of leisure time physical activity on survival after myocardial infarction has shown different survival rates associated with regular and irregular physical activity^1^. This finding suggests that the physical activity pattern may be an important factor. Presumably, personalization of physical activity patterns may facilitate rehabilitation of CVD patients, such as chronic heart failure^30^, or myocardial infarction^1^.

Comprehensive evaluation of physical activity pattern is usually accomplished relying on self-report data^2,3,6,21^. However, the recent development of electronics has given rise to more convenient means of long-term physical activity monitoring^31^ via a variety of compact, user-friendly and inexpensive devices (e.g., smart wristbands, smart watches, smartphones)^32–35^. Such devices are sufficiently accurate in tracking the number of steps^36,37^ and are becoming increasingly popular in research and clinical applications^15,17–19^.

Our findings show that larger aggregation values are expected on weekends, which is in agreement with the observations in step data collected in countries around the globe^15^. Also, we found that CVD patients assume higher aggregation values compared to healthy participants. This may be due to the fact that CVD patients spend more time in a sedentary behaviour and their physical activity of higher intensity is usually aggregated in a single session per day. The results of this study also show that females are associated with a lower aggregation value than male. This finding may be due to the fact that Lithuanian females are mostly responsible for housekeeping, in agreement with respective studies on Japanese^38^ and Brazilian individuals^39^. On the other hand, males showed higher physical activity aggregation and higher activity intensity, probably due to work-related physical activity aggregation, followed by sedentary behaviour.

Normally several days of step data are required to reliably evaluate physical activity pattern^40^. Since the proposed aggregation metric is not restricted to day-by-day analysis, the week-by-week analysis can be applied as well. The week-by-week analysis may lead to slightly lower aggregation values, since individuals are usually active at least several days per week, spreading physical activity throughout the monitoring period, and leading to decreased aggregation values. To obtain high aggregation on the week-by-week basis, most of the physical activity has to be aggregated in a single day, which is unusual, except specific patterns, such as “weekend warrior”^22^.

Low aggregation represents physical activity patterns, dominated by a combination of low intensity activity and a sedentary behaviour. Since the physical activity aggregation metric is not affected by the intensity, but rather by the pattern itself, the physical activity aggregation value is comparable for both low and high activity intensities. The current agreement is that “some activity is good, but more is better”^12,41^, thus the physical activity aggregation metric can be even more valuable if studied with respect to other metrics.

The method for quantifying temporal aggregation was first introduced to characterize the distribution of self-terminating cardiac arrhythmia episodes^42,43^. In contrast to this method, in which a single arrhythmia episode, shorter than the total monitoring period, is always assigned to a maximal aggregation, our method is flexible and duration-dependent. That is, aggregation increases when the duration of a continuous session of physical activity decreases. This update is motivated by the rationale that there is a major difference between a single very short session (e.g., 5 min) and a long one (e.g., 2 h). Therefore, it is incorrect to assign such diverse physical activity patterns to the same aggregation value.

Only a few metrics have been proposed to characterize the physical activity pattern, of which the Gini coefficient has been studied with respect to the patterns of a sedentary behaviour^4,13^ and physical activity inequality^15^. In these studies, the Gini coefficient was calculated taking into account only time periods that contain some amount of physical activity. Since the goal of the present study was to investigate the feasibility of the metrics to characterize physical activity pattern, which also embraces periods of inactivity, the Gini coefficient was calculated for the entire monitoring period. This may be the main reason of the Gini coefficient being strongly correlated with the physical activity ratio.

In this study, physical activity is defined as a number of steps per time interval. However, steps are not the characterizing descriptor for some types of physical activity, including contact sports, weightlifting, cycling, swimming. The proposed metric is not restricted to the step data and can be applied to calculate aggregation of other types of physical activities and their respective quantifiers. That is, accelerometer-based activity counts, energy expenditure, burned calories, etc., can be used to calculate the aggregation, as long as their values are sampled at sufficiently short time intervals (≤10 min).

The most widely used physical activity metrics, e.g., the number of steps per day or meeting an hourly target for specific intensity activity, are easy to interpret with an explicit goal to achieve. In case of the aggregation metric, it is unclear which value is desirable and likely depends on a specific target group a metric is applied, e.g., children, elderly individuals, patients with cardiovascular or pulmonary disease, etc. A similar problem has been encountered when interpreting other metrics, proposed to characterize the physical activity pattern, namely, the Gini coefficient^15^ and intensity gradient^16^. Therefore, future clinical studies are needed to identify the appropriate aggregation value for different population groups.

A current limitation lies on the fact that only a small fraction of smart device manufacturers provide access to the minute-by-minute physical activity data. For example, the user usually can download only the total number of steps per day, which is insufficient for computing daily or weekly aggregation. Therefore, the manufacturers could contribute to the acceleration of smart wristband research by ensuring easier access to the unprocessed data.

## Conclusions

This study introduces a physical activity aggregation metric and shows its application for an objective evaluation of the physical activity pattern. The metric can be applicable in health behavior change research, and is expected to have clinical relevance since it may provide additional information on the relationship between physical activity pattern and health outcomes, especially when used in a combination with other physical activity metrics. The metric is well-suited for implementation in smart devices capable of monitoring physical activity (smart wristbands, smartphones).

## Data Availability

The datasets recorded and synthesized in the current study are available from the corresponding author on request.
